# Effects of *Dendrobium officinale Kimura & Migo* flower flavonoids on cognitive function by regulating gut microbiota

**DOI:** 10.3389/fphar.2025.1562775

**Published:** 2025-06-26

**Authors:** Nanzhi Hu, Kaiyue Wang, Xing Ge, Xin Zhang, Xiaojie Zheng, Shifang Sun

**Affiliations:** ^1^ Department of Geriatrics, The First Affiliated Hospital of Ningbo University, Ningbo, China; ^2^ Ningbo Cadre Health Center, Ningbo, China; ^3^ Department of Food Science and Engineering, Ningbo University, Ningbo, China; ^4^ Wenzhou Vocational College of Science and Technology, Wenzhou, China

**Keywords:** *Dendrobium officinale Kimura & Migo* flower flavonoids, intestinal flora, aging, cognition, inflammation

## Abstract

**Background:**

As we get older, we experience a variety of symptoms such as memory and cognitive decline in learning. In the process of aging, neuroinflammatory response is one of the main reasons for the occurrence of cognitive dysfunction. *Dendrobium officinale Kimura & Migo* flower flavonoids (DOFF) can effectively regulate the structure of intestinal microbiota, antioxidant and anti-inflammatory functions.

**Methods:**

DOFF samples were extracted by water extraction assisted ultrasonic ethanol method. The DOFF composition was determined by LC-MS/MS method. An aging mouse model was established using D-galactose (D-gal) induced mice. 16S rDNA sequencing was used to analyze intestinal flora, hematoxylin/eosin staining (H&E) and immunohistochemical staining were used to analyze colonic and hippocampal tissue damage and related proteins, and ElISA was used to determine related inflammatory factors. The Y-maze experiment was used to test the cognitive ability of mice, the expression of related synaptic protein was detected by WB, and Iba-1 was labeled by immunofluorescence to study the effects of DOFF on inflammatory response and cognitive function by regulating intestinal microbiota.

**Results:**

The results showed that DOFF intervention could regulate the relative abundance of intestinal flora in D-gal mice, including the relative abundance of *Akkermansia*, down-regulate the ratio of *Firmicutes/Bacteroidetes,* up-regulate the expression of ZO-1, and improve the colonic tissue injury. In addition, by inhibiting the production of MDA, increasing the activity of SOD, CAT and POD, inhibiting the expression of pro-inflammatory factors TNF-α, IL-6 and IL-1β, inhibiting the activation of microglia, and effectively improving the oxidative stress damage and inflammation caused by aging. By increasing the expression of related synaptic proteins, upregulation of BDNF levels improves synaptic plasticity and alleviates cognitive dysfunction.

**Discussion:**

DOFF can improve cognitive dysfunction by regulating intestinal microbiota composition, enhancing intestinal barrier integrity in D-GAL-induced aging mice, improving neuroinflammation, and alleviating hippocampal neuron damage.

## Introduction

With the increasing aging of the population, the problems caused by aging have attracted more and more attention (Bondy et al., 2004). The brain is the most important organ in the aging process. Brain aging is characterized by widespread behavioral abnormalities, as well as phenomena such as increased oxidative stress, inflammatory responses, activation of microglia, changes in the expression of neurotrophic factors, and decreased synaptic plasticity, which together lead to the loss of neurons or neurodegeneration, leading to cognitive decline (Lupo et al., 2019; Ju and Tam, 2022). In the process of brain aging and development, pathological neurodegenerative diseases may be formed, such as Alzheimer’s disease, Parkinson’s disease, etc., causing a significant social and economic burden of neurodegenerative diseases (Spittau, 2017; Matej et al., 2019). Therefore, it is significance to explore the mechanism of brain aging and develop drugs that can effectively delay aging to prevent and treat degenerative diseases of the nervous system and improve the quality of life of the elderly (Baker and Petersen, 2018).

The balance of intestinal barrier function and gut microbes has a regulatory effect on nervous system function (Heiss and Olofsson, 2019). The human gut is colonized with a large and diverse microbial community, and its microbial composition is closely related to diseases such as aging, inflammatory bowel disease, type II diabetes, and brain cognitive dysfunction (Li et al., 2021; Zhang et al., 2022). The gut microbiota plays an important role in regulating the “gut-brain axis” bidirectional signal transduction, and changes in the gut microbiota regulate the central nervous system by regulating the formation of the blood-brain barrier (BBB), the function of microglia, and synaptic remodeling, thereby affecting learning and memory functions (Ju et al., 2023; Gan et al., 2024). Under normal physiological conditions, microglia maintain homeostasis, promote the development of neurons, the formation of tissue structures, and play a role in neuromodulation and maintenance, including promoting the formation of synaptic plasticity and the establishment of learning and memory (Mohamed et al., 2022). However, in the pathological state, the homeostasis of microglia is disturbed by neurons or other cells and signaling factors, and when over-activation occurs, inflammatory factors will be over-expressed and neurotoxic factors will be released, and long-term maintenance will lead to neurodegeneration (Azam et al., 2021). The subacute aging model of D-gal in mice is often used in anti-aging research because of its obvious aging changes and stable model. Previous studies have confirmed that the aging model made by D-gal shows similar changes to natural aging in many morphological, physiological and biochemical indicators of various organs and tissues (Shwe et al., 2018). Age-related changes in the composition of the gut microbiota include a decrease in the diversity of the microbiota, a decrease in the abundance of beneficial microorganisms, an increase in the abundance of potential pathogenic bacteria, resulting in an imbalance in the proportion of microbiota (Du et al., 2021). The balance of intestinal flora affects the permeability function of the intestinal barrier, and lipopolysaccharide (LPS), a metabolite of the cell wall of gram-negative bacteria, enters the blood circulation when the tight junction of the intestinal barrier is disrupted, triggering an inflammatory response (Yu et al., 2020). It has been found that intraperitoneal injection of LPS in mice can cause neuroinflammation and lead to learning and memory impairment along with Aβ deposition in the hippocampus (Khan et al., 2019). At the same time, the inflammatory signals produced by dysfunctional peripheral macrophages in the brain brought about by aging are transmitted to microglia through the highly permeable BBB, which accumulates over time and gradually impairs the body’s cognitive function (Preininger and Kaufer, 2022). Regulates the composition and optimal function of the gut microbiota and affects the activation of glial cells and the expression of pro-inflammatory factors through conduction between the gut-brain axis, and improves the cognitive impairment caused by neuroinflammation (Song et al., 2024). Therefore, the balance of intestinal microbiota plays an important role in protecting the integrity of the intestinal barrier and inhibiting the occurrence of neuroinflammation and cognitive impairment.

With age, hippocampal synaptic plasticity declines, resulting in cognitive decline (Català-Solsona et al., 2021). Hippocampal synaptic plasticity involves many synapse-related proteins, neurotransmitters and other substances, among which brain-derived neurotrophic factor (BDNF). Synaptophysin (SYP) and postsynaptic density 95 (PSD95) are recognized as important substances related to cognitive ability in the hippocampus, which play an important role in synaptic connection and shaping (Liu et al., 2021). Neurotrophic factor is a secreted protein that plays a major role in synaptic and neuronal growth, myelination, differentiation, and survival. Compared with other neurotrophic factors, BDNF is more widely distributed and expressed in large numbers in brain regions closely related to learning and memory, such as hippocampus, cortex, cerebellum (Numakawa and Kajihara, 2023). BDNF mainly regulates synapse-related proteins by regulating neuronal plasticity and binding to a variety of receptors, thereby affecting learning and memory (Lu and Figurov, 1997). In addition, BDNF not only plays a role in the development and maturation of neurons in normal physiological processes, but also plays a substantial role in protecting, repairing, and promoting regeneration when pathological changes occur in the nervous system, and has great potential application value in drug research and development in the treatment of neurodegenerative diseases and psychiatric diseases (Liu and Wang, 2020).

Dietary interventions may be the factors most directly influencing gut function, particularly the microbiota (e.g., *Firmicutes* and *Bacteroidetes*) (Yang et al., 2019). It may mediate conduction between the enteric nervous system and the central nervous system by regulating the composition and optimal function of the gut microbiota, thereby improving glial cell activation and, in turn, neurological inflammation (Gan et al., 2024; Guo et al., 2019). With the occurrence and development of aging, the body’s ability to scavenge free radicals produced in the body decreases, and the accumulation of reactive oxygen species (ROS) in cells will directly damage the function of biological macromolecules such as mtDNA, proteins and lipids in cells, further accelerating the aging process, and the accumulation of long-term free radicals will promote the body to be in a state of chronic inflammation (Zia et al., 2022). Flavonoids are widely present in a variety of natural plants, which have pharmacological effects such as antioxidant, anti-aging, antitumor, antibacterial, anti-inflammatory, anti-cancer and regulating vascular penetration (Sharifi-Rad et al., 2022a). Studies have found that the effects of flavonoids in traditional Chinese medicine on the nervous system include protecting neurons from neurotoxin damage, inhibiting neuroinflammatory responses, alleviating oxidative stress damage, regulating various cell signaling pathways, and promoting memory, learning and cognitive functions (Song, et al., 2024; Spencer, 2009). In addition, flavonoids can also improve the body’s inflammatory response by reversing the abnormal abundance of gut microbiota and regulating metabolites, reducing disturbed levels of neurotrophic factors, neurotransmitters, and stress-related hormones (Wang et al., 2023).

*Dendrobium officinale Kimura & Migo* is a perennial epiphytic botanical drug of the genus Dendrobium of the orchid family, which has the effects of nourishing the stomach, the lung and the kidney (Chen et al., 2021). Until 2023, a total of 261 compounds have been extracted, separated and identified from *Dendrobium officinale Kimura & Migo*, which can be divided into flavonoids, bibenzylates, alkaloids, phenylpropanoids and other components according to their structure. Because *Dendrobium officinale Kimura & Migo is* a resource for both medicine and food, its chemical components are mainly concentrated on stems, leaves and flowers, and flavonoids, bibenzyl and phenylpropanoids are considered to be the main components (Meng et al., 2023; Yang, et al., 2022). *Dendrobium officinale Kimura & Migo* is widely used because of its high medicinal value and perfect large-scale planting, and as a top Chinese medicine, it has no adverse reactions reported so far, indicating its edible safety and reliability. The current distribution of major industries and product characteristics are shown in Tables 1, 2 (Hu et al., 2024; Ni et al., 2023).

**TABLE 1:** Product characteristics of *Dendrobium officinale Kimura & Migo*.

1. Characteristic	Stem erect, cylindrical, 9–35 cm long, 2–4 mm thick, unbranched, multinode; leaves disserial, papery, oblong-lanceolate, margin and midrib often lilac
2. Well characterized	
Active ingredients known	Flavonoids, Bibenzylates, Alkaloids, Phenylpropanoids (according to structure)
Specific classification of active ingredients is known	Flavonoids: Apigenin, Tangeretin, Apigenin-6-C-a-L-arabinose-8-C-β-D-xylose, Apigenin-6-C-a-L-rhamnose-8-C-β-D.xylose, SchaftosideFlavonoids, rhamnose, Isoschaftoside Bibenzylates: Dendrocandin A-R, 3-Methoxystrobinol, Batatasin Ⅱ, Dendrosinen B, Moscatilin etc. Alkaloids: Dihydro-fe ruloyltyram ine, N-(cis-Feruloy!)tyramine, Spermidine, 2-Benzothiazolol etc. Phenylpropanoids: 4-Hydroxycinnamicacid, (Z)-4-Hydroxycinnamicacid, Femulie Acid, Chlorogenic acid, Syringin etc.
3. Free of adulteration and contamination	
4. Stable	

**TABLE 2:** Main industries of *Dendrobium officinale Kimura & Migo* (2002–2023) (Hu, et al., 2024).

Cultivation	47.26%
Pharmaceutical preparation	17.20%
Beverage industry	9.96%
Tea products	8.38%
Cosmetics	8.10%
Tissue culture	6.90%
Nutraceutical preparation	2.20%

At present, the research on *Dendrobium officinale Kimura & Migo* mostly focuses on polysaccharides and alkali metabolites, and there are few studies on flavonoids (Chen et al., 2018). Studies have shown that the flavonoids of *Dendrobium officinale Kimura & Migo* have the effects of lowering blood sugar, regulating blood lipids, antioxidant and anti-pathogenic microorganisms, can scavenge free radicals, have good anti-aging effects, and are potential therapeutic drugs for the prevention and treatment of cognitive dysfunction (Zhang et al., 2023). It has been found that *Dendrobium officinale Kimura & Migo* flower had antioxidant activity comparable to that of soybean isoflavone extract, a traditional flavonoid with strong antioxidant activity. Pharmacological studies have pointed out that *Dendrobium officinale Kimura & Migo* and its active metabolites have anti-inflammatory properties, which can be achieved by regulating the production and release of cytokines related to inflammatory responses, such as interleukin-6 (IL-6), interleukin-1β (IL-1β) and tumor necrosis factor-α alpha (TNF-α) (Fan et al., 2024). The study found that quercetin significantly inhibited the mRNA expression of TNF-α and interleukin-1α (IL-1α) stimulated by LPS in the environment of glial cell-neuronal co-culture, suggesting that quercetin could reduce inflammatory apoptosis of neuronal cells (Wang et al., 2020). Animal experiments have found that quercetin may alleviate oxidative stress and inflammation in model rats by inhibiting the NF-κB pathway and epidermal growth factor receptor (EGFR) phosphorylation (Li et al., 2022). Based on the above research background, in this study, D-galactose (D-gal) was used to establish an aging mouse model, and the ameliorating effect of *Dendrobium officinale Kimura & Migo* flavonoids (DOFF) on D-gal-induced intestinal barrier damage and hippocampal neuronal system damage in aging mice by regulating gut microbiota was investigated through *in vivo* experiments.

## Materials and methods

### Materials and reagents

*Dendrobium officinale Kimura & Migo* flowers were originated from Zhejiang Province, China. L-ascorbic acid, ELISA analysis of biochemical parameters of D-galactose (Sigma), including LPS, IL-6, IL-1β, malonaldehyde (MDA), superoxide dismutase (SOD), catalase (CAT), and peroxidase (POD) were purchased from Shanghai Biotechnology Co., Ltd. (Shanghai, China). All other reagents and chemicals were analytical-grade reagents.

### Preparation of DOFF

Fresh *Dendrobium officinale Kimura & Migo* flowers were dried to a certain weight and ground into a powder (80 mesh sieve). *Dendrobium officinale Kimura & Migo* flower powder was taken, 70% ethanol was added according to the solid-liquid ratio of 1: 52 (g/mL), the ultrasonic time was set for 35 min, the ultrasonic power was 180 W, and the temperature was 40 °C. After the end of ultrasonication, the sample solution was placed in a water bath, the water bath temperature was set at 55 °C, the water bath duration was 2 h, the extraction was repeated twice, the filtrate was mixed, vacuum filtered, and concentrated to a certain volume with a rotary evaporator. The concentrate was added to 2 times the volume of absolute ethanol at 4 °C overnight, the filter residue was removed, and the extract was spun and steamed again to the extract state.

AB-8 macroporous resin was wet packed into columns, the solid-liquid ratio of resin to sample solution was 1:1.74, More than 95% ethanol was added to the chromatography column 10 cm higher than the resin layer for 4 h, and then rinsed with distilled water until the effluent was diluted with water in the test tube and was not turbid. Finally, wash repeatedly with water until there is no obvious ethanol odor. The final water level should be kept above the resin level to avoid drying out the column.

1.5 g of total flavonoids of *Dendrobium officinale Kimura & Migo* leaves were weighed, and 2.2 mg/mL loading solution was prepared with water, with a loading rate of 4 BV/h and a sample loading amount of 16 BV. Then elution with water to remove impurities, the rate is 4 BV/h, and the dosage is 3 BV. This was followed by elution with 80% ethanol at a rate of 1 BV/h at a dosage of 3.25 BV. Collect the eluate. DOFF powder is obtained after drying under reduced pressure.

### DOFF quantitative analysis

The DOFF was analyzed using the UHPLC-MS/MS method. The analysis was performed using a Vanquish UHPLC system (Thermo Fisher, Germany) coupled with a Orbitrap Q Exactive TM high-frequency mass spectrometer (Thermo Fisher, Germany). Liquid phase conditions: 0.1% formic acid-water (A); 0.1% formic acid-acetonitrile (B). The gradient elution was set to 98% (A), 2% (B), 0–2 min; 100% (A), 2–17 min; to 98% (A), 2% (B), 17–20 min. Chromatographic conditions: the column adopts Hypesil Gold column (1002.1 mm, 1.9 μm) column temperature of 40 °C, flow rate of 0.2 mL/min, sample flow rate of 0.2 mL/min, injection volume of 20 µL. Mass spectrometry conditions: HESI ion source, negative ion detection mode, Full MS/dd-MS2 scan mode, Full MS resolution 70,000, dd-MS2 resolution 17,500, and scan range 100–1,500 m/z. Q Exactive TM HF The mass spectrometer operates in positive and negative polarity mode with spray voltage of 3.2 kV, capillary temperature of 320°C, sheath gas flow rate of 40 arb and auxiliary gas flow rate of 10 arb. Mass spectrometry data was processed using CD software to match possible molecular configurations in the mzCloud, mzVault and masslist local databases according to the MS data of the sample metabolites.

### Animal experiments

C57BL/6J male mice (6–8 weeks old, 20 ± 2 g) were housed in SPF animal houses with alternating light-dark for 12 h, temperature 22 ± 2°C, humidity 65% ± 5% (Murayama et al., 2021; Tucker et al., 2016). Mice were free to drink and eat throughout the experiment, and our research protocol was designed to minimize the number of animals used and their suffering. Our research protocol has been approved by the Laboratory Animal Care Ethics Committee of Ningbo University (license number: SYXK [Zhejiang] 2019–0001). After 1 week of acclimatization, the mice were randomly divided into 4 groups, including blank group (CK), aging model group (CD), positive control group (VC), and sample group (DOFF), with 15 mice in each group. The CK group was intragastric with 0.2 mL of sterile saline, and 0.2 mL of sterile saline was injected subcutaneously at the back of the neck. In the CD group, mice were given 0.2 mL of sterile normal saline, and 0.2 mL of D-gal solution (500 mg/kg/d) was injected subcutaneously into the hinderior neck of mice according to the previously reported method to establish an aging model (Chen et al., 2016; Liu et al., 2023). The DOFF group was given 0.2 mL of DOFF solution (200 mg/kg/d) by gavage, and 0.2 mL of D-gal solution (500 mg/kg/d) was injected subcutaneously in the back of the neck (Duan et al., 2017). All gavage and subcutaneous injections continued for 7 weeks and were administered at regular intervals each day.

### Determination of DOFF dose in animal experiments

Based on published experimental studies, we first calculated a safe starting dose for human clinical trials using the content of DOFF. The animal drug dose is then estimated by a scale factor (human clinical dose: animal dose = 1: 33), and the human clinical dose is converted to an animal dose (Nair et al., 2018). Therefore, we determined that the appropriate DOFF dose is 200 mg/kg/d.

### Sample processing and collection

On the last day of gavage, stool samples are collected using a sterile Eppendorf tube and immediately stored at −80°C for analysis of the gut microbiota. 24 h before the collection of brain tissue and serum samples, the mice were fasted and watered overnight, and then anesthetized with intraperitoneal injection of 4% chloral hydrate (400 mg/kg), and the eyeballs were collected after complete anesthesia. Immediately after blood collection, centrifuge at 3,000 r/min for 20 min at room temperature, aspirate serum and divide appropriately, and store in a −80°C freezer for testing. Immediately after the blood collection, the mice were denecked, and the mice were perfused with 4% paraformaldehyde (formalin) in the heart, thereby directly separating the whole brain from the ventricles, and then separating the hippocampus and cerebral cortex. Portion of the hippocampus and cerebral cortex are fixed in preprepared 4% paraformaldehyde; The remainder was quick-frozen in liquid nitrogen and stored in a −80°C ultra-low temperature freezer for later use. Strict care should be taken throughout the procedure to avoid damaging the integrity of brain tissue.

### Western blot

Twenty mg of brain tissue was dissolved in 100 μL lysate, and centrifuged, and part of the supernatant protein was determined with BCA protein concentration assay kit. According to the molecular weight of the protein, the lower and upper layers of the gel are arranged and a comb is inserted. Samples are loaded into sample wells, separated by electrophoresis, and transferred to PVDF membranes. After transfer, block the PVDF membrane with 5% BSA at room temperature and wash with TBST. PSD95 (protein 1:1000 rabbit source), SYP (protein 1:4,000 rabbit source), and β-actin (antibody 1:2000 mouse source) were diluted with a blocking solution and incubated overnight at 4 °C. After washing the membrane with TBST, horseradish peroxidase (HRP)-labeled goat anti-mouse secondary antibody (antibody 1:2000) is incubated for 2 h at room temperature. The cell membrane is removed and color is developed. The ChemiDox Gel Imaging System was imaged and Quantity One Software was used for grayscale analysis of image acquisition bands and protein bands, using the intensity of the corresponding β-actin bands as a standard.

### Y maze

Y maze test method is a kind of behavior, designed to evaluate the animal of working memory and recognition ability of learning (Hamieh et al., 2021). The spatial memory and decision-making strategies of the animals can be analyzed by recording in detail the order in which the animals entered each arm of the maze, the time spent, and the number of correct and wrong choices (Ghafarimoghadam et al., 2022). All mice involved in the experiment were allowed to freely explore the Y maze for 8 min after dark adaptation for 0.5 h. During the experiment, the mice’s movements were captured using specialized behavioral cameras. By recording the number and order of the mice entering each arm, the correct rate of spontaneous alternations was calculated. The calculation formula of spontaneous alternations correct rate is: spontaneous alternations correct rate = number of correct spontaneous alternations/(total number of inlet arm −2) × 100%. In the Y-maze test, the paths entered by the first two times were ignored and calculated from the third time. If the path chosen by the animal was different from the previous two times, it was considered to be the correct path choice and recorded as a correct spontaneous alternation. If the animal’s path selection is the same as on either of the previous two occasions, it is considered wrong path selection.

### Immunofluorescence assays

In immunofluorescence experiments, hippocampal tissues fixed with 4% paraformaldehyde were removed and subjected to a series of dehydration, paraffin-embedded, and cut into sections (4 μm). Hippocampal tissue (4 μm) sections were removed with xylene, rehydrated with different concentrations of ethanol, and washed with PBS solution. Slides were immersed in EDTA antigen retrieval buffer (pH 8.0) for antigen retrieval. Add 3% BSA to cover the labeled tissues and block non-specific binding for 30 min. The slides were incubated with anti-Aβ (Hangzhou Dating Biotechnology Co., Ltd.) overnight at 4°C, followed by secondary antibodies for 50 min. Nuclei were stained with 4′, 6-diamino-2-phenyllindol (DAPI). Microscopic inspection and image acquisition were performed using a fluorescence microscope. Fluorescence intensity was calculated using ImageJ software.

### H&E staining and immunohistochemistry

Fresh colon and hippocampal tissues were partially dissected, fixed with 4% paraformaldehyde for 24 h, dehydrated, and embedded in paraffin, the wax block was cut into thin slices with a microtome, flattened on the water surface at 42°C, and the slides were fished out with APES-coated slides, and inserted into the slicing rack and dried in a 37 °C incubator. Sections are cooled, H&E stained, sealed and placed under an inverted microscope for observation and photographing. The histologic scoring system was shown in Table 3.

**TABLE 3:** The histologic scoring system.

Scoring criteria (points)	0	1	2	3
Inflammatory cell infiltration	Nothing	Mild	Moderate	Severe
Blood loss	Nothing	Mild	Moderate	Severe
Submucosal edema	Nothing	Mild	Moderate	Severe
Intestinal mucosal surface integrity	Good	Small cup-shaped or epithelial cell loss	Cup cells are often missing and the epidermis is damaged	The entire crypt and epidermis are damaged

Immunohistochemical staining: Frozen sections were air-dried at room temperature, and baked in a 37°C oven, fixed in methanol and washed with PBS (pH 7.4) on a decolorizing shaker. Add EDTA (pH 9.0) to microwave and boil the antigen retrieval solution, soak in 3% hydrogen peroxide solution, and incubate at room temperature in the dark. The slides were placed in PBS solution (pH 7.4) and washed by shaking on a decoloring shaker. Shake the sections dry, draw a circle around the target tissue, add drops, cover the tissue evenly with 3% bovine serum albumin, and block for 30 min at room temperature. Incubate overnight with primary antibody, add secondary antibody, and incubate at room temperature for 50 min. DAB colorant is used for color development, and the degree of staining is controlled under the microscope. Deionized water terminates the chromogenic reaction and rinses well. Hematoxylin was retained, fully rinsed with double distilled water, hydrochloride alcohol identification, gradient alcohol conventional dehydration, xylene transparent, neutral glue sealed, and resin mount observed on an optical microscope.

### ELISA

Collected cerebral cortex (indicated amount) homogenized, centrifuged. The levels or activities of LPS, MDA, SOD, CAT, and POD in serum, as well as the levels of IL-6, IL-1β, TNF-α in the cerebral cortex and serum were detected by the kit. The ELISA performed the experiment according to the instructions procedure. According to the established standard curve, the raw OD value was converted to the sample concentration value, and the data were collated and analyzed.

### Data analysis

A randomized group design was used, with at least three replicates in each group. GraphPad Prism software V.9.5. One-way ANOVA was performed and Tukey’s multiple comparison test was performed. *P* < 0.05 indicated that the difference was statistically significant (*p < 0.05, **p < 0.01, ***p < 0.001, ****p < 0.0001).

## Results and discussion

### DOFF quantitative analysis results

**FIGURE 1:**
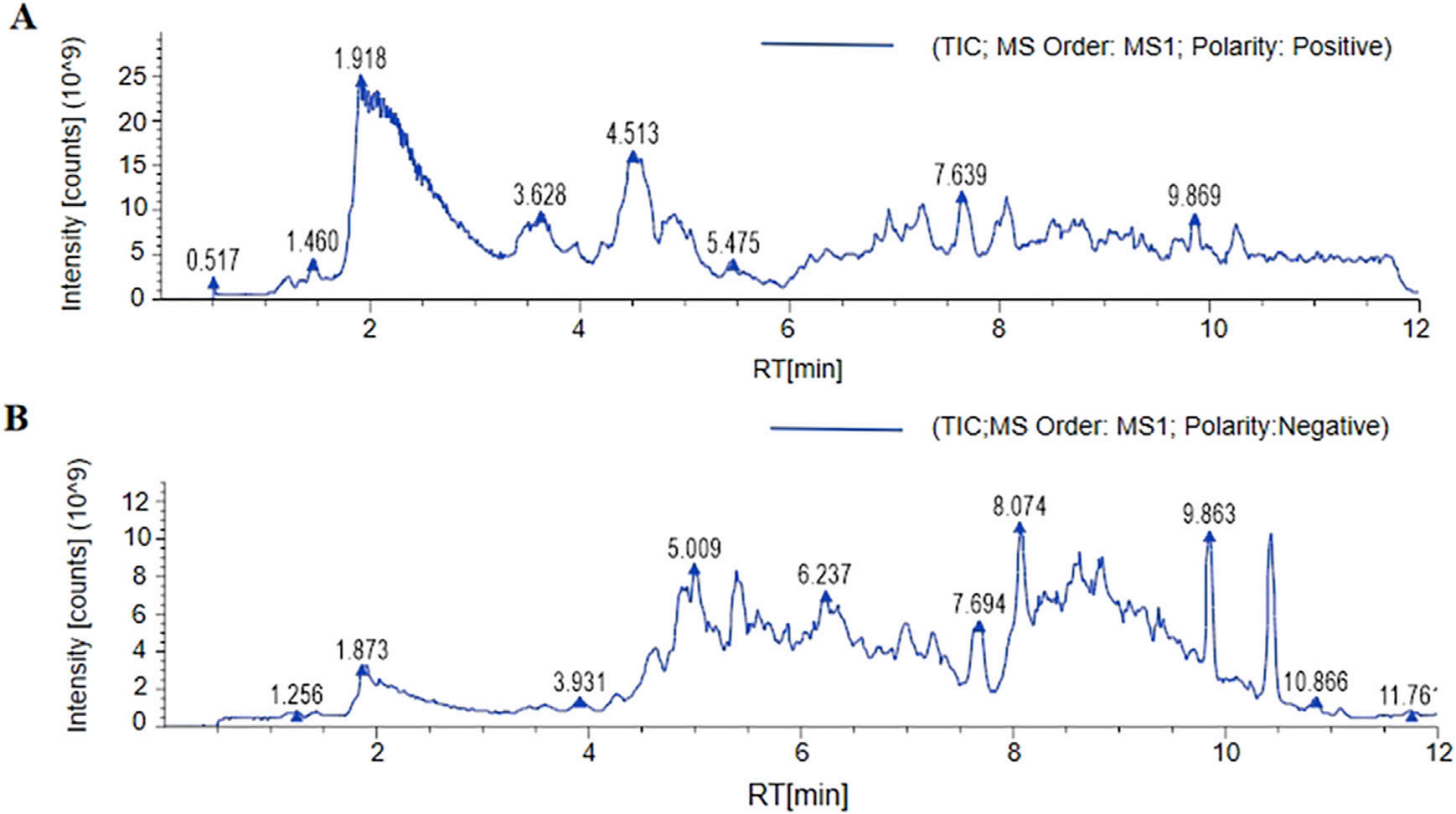
The component analysis of DOFF. **(A)** LC-MS/MS positive ion flow diagram. **(B)** LC-MS/MS negative ion flow diagram.

The chemical composition of the DOFF was analyzed using UPLC-Q-Exactive Orbitrap-MS technology. Fifty chromatographic peaks in DOFF (Figures 1A,B) were identified by scanning and extracting the ion peaks by complete one-stage mass spectrometry, and the chemical composition of the DOFF alcohol extract was synthesized and comprehensively identified by combining retention time, fragmentation information, and database synthesis. The relative quantification of the top 25 substances in DOFF was shown in Table 4. Among them, the main active metabolites are Isoschaftoside, Myricitrin, Schaftoside, Apigenin-6,8-di-C-glycoside, Hesperetin 5-*O*-glucoside, and Tulipanin, Glucosylvitexin, etc. Among them, flavonoids and flavonols accounted for most of the identified substances, followed by flavanones, anthocyanins, and isoflavones (Deng et al., 2024; Yuan et al., 2022). Studies have shown that flavonols can improve cognitive dysfunction in experimental animals by inhibiting Aβ production and aggregation, inhibiting Tau protein phosphorylation, restoring synaptic transmission, and restoring the level of brain-derived neurotrophic factors (Horvat et al., 2023). In addition, flavonols protect the gut microbiota and can also improve intestinal barrier function by increasing the expression of butyric acid receptors and connexins in the intestinal mucosa (Fan et al., 2022; Zhao et al., 2023). These results suggested that DOFF has the effect of regulating intestinal microbiota and improving cognitive impairment of the nervous system.

**TABLE 4:** LC-MS quantification of DOFF, resulting from the relative quantification of the top 25 substances in DOFF.

Name	Formula	Molecular Weight	RT [min]	Class
Isoschaftoside	C_26_H_28_O_14_	564.499	6.03	Flavones and Flavonols
Myricitrin	C_21_H_20_O_12_	464.38	6.547	Flavones and Flavonols
Schaftoside	C_26_H_28_O_14_	564.492	6.074	Flavones and Flavonols
Apigenin-6,8-di-C-glycoside	C_27_H_30_O_15_	594.518	5.73	Flavones and Flavonols
Hesperetin 5-*O*-glucoside	C_22_H_24_O_11_	464.419	6.59	Flavanones
Tulipanin	C_27_H_31_O_16_	611.5	6.35	Anthocyanins
Glucosylvitexin	C_27_H_30_O_15_	594.526	6.25	Flavones and Flavonols
C- pentosyl-C-D- hexosyl-apigenin	C_26_H_28_O_14_	564.49	6.05	Flavones and Flavonols
Kaempferol-3-*O*-rutinoside	C_27_H_30_O_15_	594.526	6.51	Flavones and Flavonols
Tricin 5-*O*-hexoside	C_23_H_24_O_12_	492.43	7.19	Flavones and Flavonols
Tricin *O*-malonylhexoside	C_26_H_26_O_15_	578.47	7.5	Flavones and Flavonols
Isorhamnetin-3-*O*-neohespeidoside	C_28_H_32_O_16_	624.552	6.79	Flavones and Flavonols
Kaempferol 7-*O*-beta-D-glucopyranoside	C_21_H_20_O_11_	448.377	6.65	Flavones and Flavonols
Chrysoeriol C-hexoside	C_22_H_22_O_11_	462.404	6.72	Flavones and Flavonols
C-hexosyl-luteolin *O*-hexoside	C_27_H_30_O_16_	610.518	5.68	Flavones and Flavonols
Myricetin 3-*O*-galactoside	C_21_H_20_O_13_	480.376	7.19	Flavones and Flavonols
C- pentosyl-luteolin-D- C-hexoside	C_26_H_28_O_15_	580.49	6.38	Flavones and Flavonols
Tricin	C_17_H_14_O_7_	330.289	7.5	Flavones and Flavonols
C-pentosyl-apigenin *O*-p-coumaroylhexoside	C_35_H_34_O_16_	710.63	7.17	Flavones and Flavonols
Chrysoeriol 5-*O*-hexoside	C_22_H_22_O_11_	462.404	7.18	Flavones and Flavonols
Tricin 5-*O*-hexoside derivative	C_24_H_22_O_14_	534.42	7.67	Flavones and Flavonols
Pratensein-7-*O*-glucoside	C_22_H_22_O_11_	462.41	6.86	Isoflavonoids
Quercetin-3,4′-*O*-di-beta-glucopyranoside	C_27_H_30_O_17_	626.52	5.39	Flavones and Flavonols
Naringenin chalcone	C_15_H_12_O_5_	272.253	8.518	Chalcones and dihydrochalcones
Azaleatin	C_16_H_12_O_7_	316.265	7.3	Flavones and Flavonols

### Effects of DOFF on blood LPS levels and intestinal barrier integrity in aging mice

**FIGURE 2:**
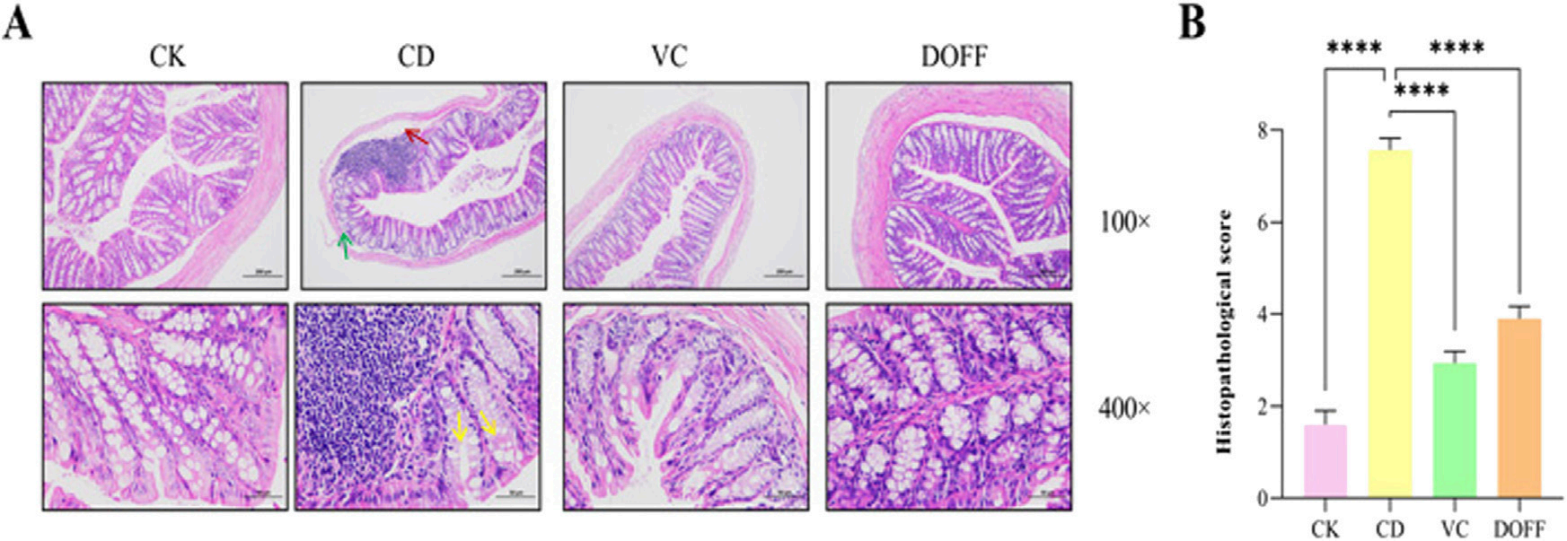
Statistical results of HE staining of colon tissue and degree of injury. **(A)** H&E staining of mouse colon; **(B)** Histopathological score. Data are presented as mean ± SEM, n = 15; *p < 0.05, **p < 0.01, ***p < 0.001, ****p < 0.0001, compared with the CD group.

In order to study the effect of DOFF on the colonic barrier, H&E staining was used to observe the morphology, inflammatory cell infiltration, and goblet cell number of colon crypts. The results of H&E staining showed that the mice in the CD group had extensive histopathological lesions and in the CD group, vasodilation, hemorrhage, colonic mucosal and submucosal edema, muscle thickening, mucosal epithelial cell degeneration, and goblet cell decrease. As shown in Figure 2A, the CD group exhibited vasodilation and hemorrhage, thickening of the muscle layer, and edema of the colonic mucosa and submucosa (red arrow); subsequently, there was degeneration and shedding of the epithelial cells in the mucosal layer, as well as loss of goblet cells (yellow arrow). We have also observed a significant increase in the number of infiltrating lymphocytes in the CD group (green arrow). However, compared to the mice in the CD group, the histological changes of colon cells in the DOFF group were improved, there was no significant swelling, and the rupture of the epithelial layer was repaired. Pathological scores showed improvement in tissue damage compared to the CD group (*p* < 0.0001, Figure 2B). These results revealed that aging may damage the integrity of the intestinal barrier and cause inflammatory responses, and DOFF can alleviate the pathological damage and inflammatory responses caused by aging to a certain extent.

**FIGURE 3:**
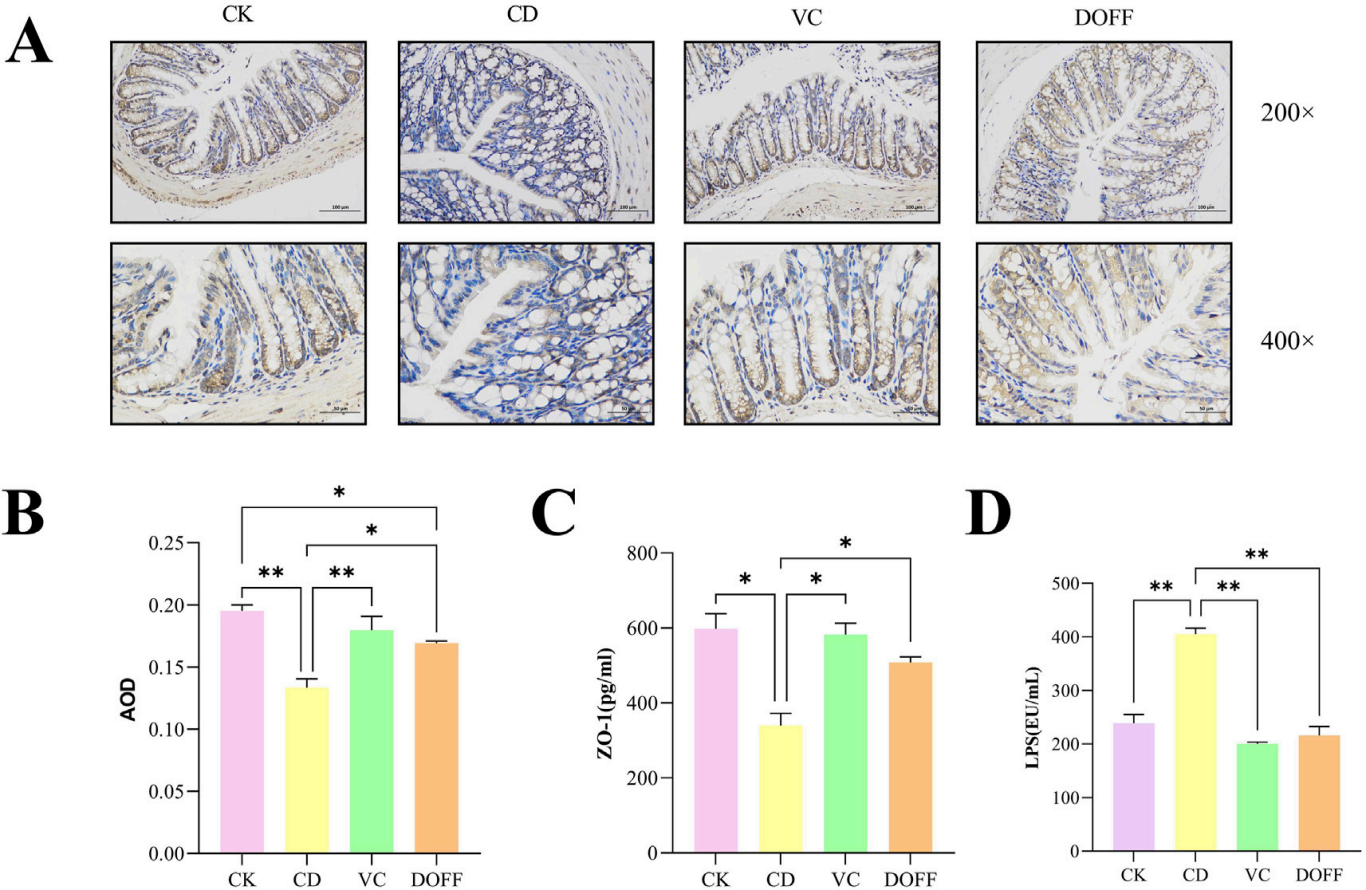
Effects of DOFF on blood LPS levels and intestinal barrier integrity in aging mice. **(A)** Immunohistochemical staining of tight junction protein ZO-1 in mouse colon tissue; **(B)** AOD value of tight junction protein ZO-1; **(C)** ZO-1 content in colon tissue; **(D)** LPS level in serum. Data are presented as mean ± SEM, n = 15; *p < 0.05, **p < 0.01, ***p < 0.001, ****p < 0.0001, compared with the CD group.

The intestinal barrier is the first line of defense to protect the body from LPS damage, which can cause inflammatory factors such as LPS produced by the intestine to enter the bloodstream and cause a systemic inflammatory response (Li et al., 2020). As shown in Figure 3D, the level of LPS in the DOFF group was significantly lower than that in the CD group (*p* < 0.01). In addition, studies have shown that LPS can induce peripheral and cerebral inflammatory responses, which suggest that DOFF may improve the inflammatory response during aging, possibly by reducing the level of LPS in the blood.

A large number of lymphoid tissues and cells are distributed between the epithelial cells, lamina propria, and submucosa of the intestinal mucosa, constituting the intestinal immune barrier. Intestinal epithelial cells are tightly connected, modifying the integrity of the intestinal barrier and forming a physical barrier. It is the first line of defense against the invasion of harmful pathogens and luminal substances (Awad et al., 2017). To further confirm the regulation of intestinal barrier function by DOFF, we tested the expression of ZO-1 in colon tissue. The positive expression of ZO-1 was brownish-yellow, and the average optical density (AOD) value of each protein expression was calculated by ImageJ software, and quantified by ELISA. The results (Figures 3A,B) showed a significant decrease in ZO-1 expression compared to the CD group (*p* < 0.05). At the same time, the results of ELISA analysis (Figure 3C) showed a significant increase in ZO-1 (*p* < 0.05) after DOFF intervention compared to the CD group. Therefore, the results suggest that the effect of DOFF on intestinal barrier function can improve the intestinal mucosal damage caused by aging by upregulating the expression of ZO-1.

The disruption of tight junction proteins impairs the integrity of the BBB, allowing neurotoxic substances to enter from the periphery, leading to the development of neuroinflammation and neurodegeneration (Lochhead et al., 2017; Cohen et al., 2024). Interestingly, we found that the imbalance of gut microbiota was significantly improved after DOFF intervention. Through the analysis of DOFF, it can be seen that the content of the substances is mainly flavonoids and flavonols. Existing studies have proved that flavonol supplementation can regulate the expression of intestinal mucosal receptors and connexins and increase their number, thereby effectively protecting intestinal health (Endo et al., 2019).

### Effect of DOFF on the structural composition of intestinal microbiota in aging mice

**FIGURE 4:**
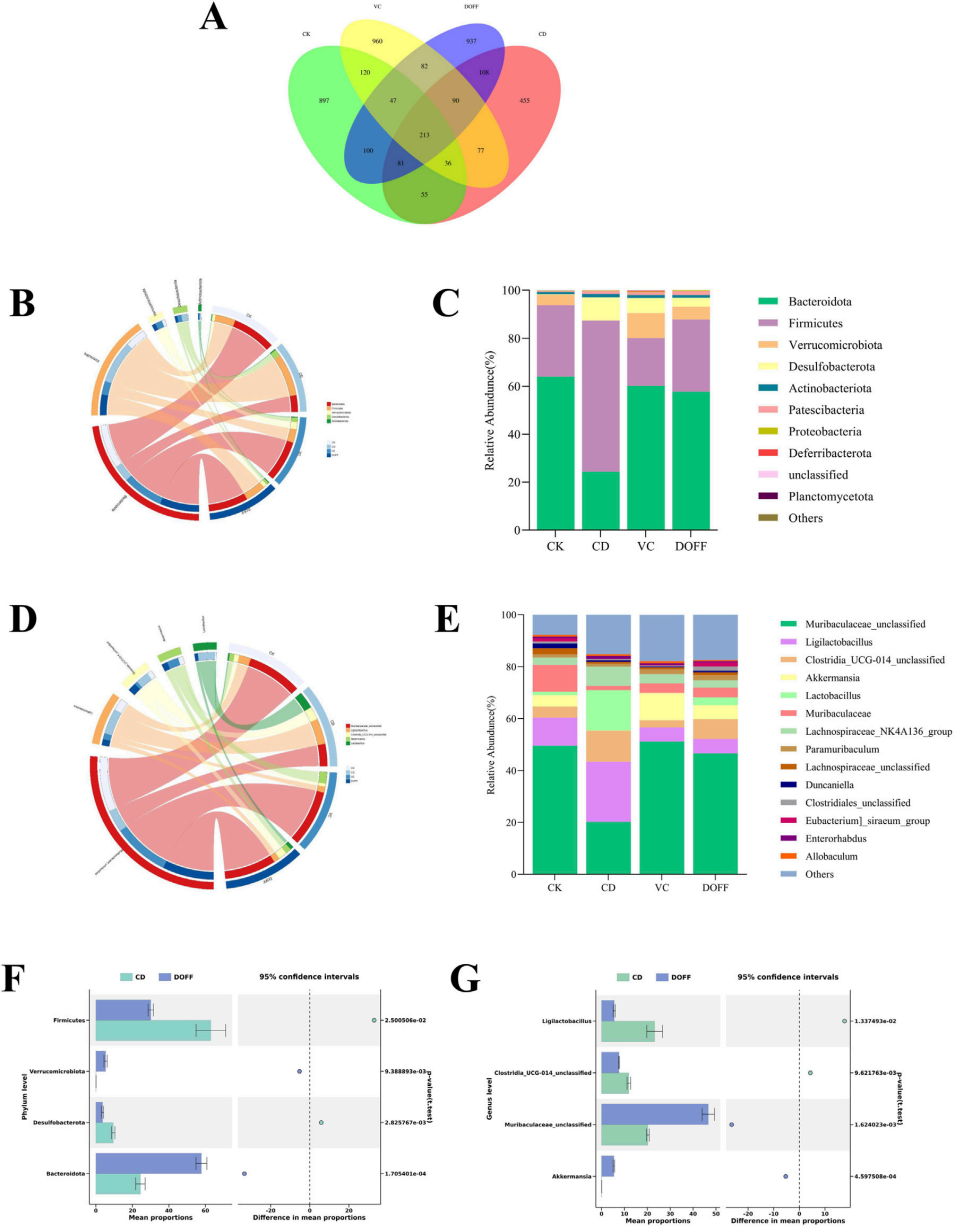
Effect of DOFF on the structural composition of intestinal microbiota in aging mice. **(A)** Venn diagram of ASV distribution; **(B)** horizontal circle diagram of phylum of intestinal flora; **(C)** difference in composition of intestinal flora of mice in the four groups on the phylum level; **(D)** horizontal circle diagram of genus of intestinal flora; **(E)** difference in composition of intestinal flora of mice in the four groups on the genus level; **(F)** difference in composition of CD and DOFF at phylum level; **(G)** difference in composition of CD and DOFF at genus level. Data are presented as mean ± SEM, n = 15; *p < 0.05, **p < 0.01, ***p < 0.001, ****p < 0.0001, compared with the CD group.

With the aging of the body, the composition of microorganisms in the intestine will change, and the dynamic balance of the intestinal flora will be broken, which is manifested as a decrease in the diversity of microorganisms, a decrease in the number of bifidobacteria, and an increase in the number of facultative anaerobes (Kim et al., 2021). Therefore, to determine the effect of DOFF on the changes in intestinal microbiota, we collected fecal samples from mice in each group and further detected the changes in the structure of intestinal microbiota in each group by genome sequencing. The Venn diagram shows the OTUs and overlaps for each group. The Venn diagram (Figure 4A) shows that there were 1549, 1115, 1625, and 1658 OTUs for CK, CD, VC, and DDFF, respectively, with 213 OTUs shared by the four groups and 897, 455 and 897, 455 and 855 OTUs unique to each group, respectively 960 and 937 pcs. The relative abundance of bacteria indicates the structure and stability of the intestinal microenvironment. Overall, the four most abundant phyla include *Bacteroidetes*, *Firmicutes*, *Verrucomycetes*, and *Proteobacteria* (Figures 4B,C). Compared with the CD group, the relative abundance of *Firmicutes* and *Proteobacteria* in the DOFF group decreased significantly (*p* < 0.05), and the relative abundance of *Proteobacteria* decreased significantly (*p* < 0.01), while the relative abundance of *Bacteroidetes* increased (*p* < 0.001) and the F/B ratio was also significantly decreased (0.52 and 2.58), respectively (Figure 4F). Studies have confirmed that an increase in the ratio of *Firmicutes/Bacteroidetes* is an indicator of aging. These results indicated that DOFF inhibited *Firmicutes*, contributed to the proliferation of *Bacteroidetes*, played a positive role in regulating intestinal microecology, and played a role in delaying aging. In addition, there is an increase in some potentially pathogenic bacteria, such as *Proteobacteria*. As can be seen from the results, compared with the CD group, the relative abundance of *Proteobacteria* in the intestine in the DOFF group decreased (*p* < 0.01). Studies have shown that the abundance of verrucous micro bacteria is closely related to gut health, contributes to glucose homeostasis in the human gut, and has anti-inflammatory properties. The abundance of *verrucous microphyta* in the CD group was significantly lower than that in the DOFF group (*p* < 0.01). It is shown that DOFF helps to reduce the abundance of pathogenic bacteria in the intestine, which is conducive to the growth of microorganisms with anti-inflammatory properties, and further contributes to intestinal health. DOFF can be used as a prebiotic to promote the proliferation of specific gut microbiota, primarily including *Akkermansia* and *Parabacteroides*. *Akkermansia musiniphila,* a mucin-degrading bacterium belonging to *Verrucomicrobia*, colonizes the intestinal mucus layer and improves the integrity of the intestinal barrier by promoting mucin production (Hu et al., 2022; Cui et al., 2022).

To further characterize the differences in gut microbiota in mice under different treatments, we performed statistical analyses of the relative abundance of microbiota in the samples at the genus level (Figures 4D,E). After the induction of senescence and the intervention of DOFF, the structure of the intestinal microbiota of mice was significantly changed. The results (Figure 4G) showed that the relative abundance of *Akkermansia* and *Muribaculaceae_unclassified* in the DOFF group was significantly higher than that in the CD group (*p* < 0.001, *p* < 0.01). Studies have confirmed that *Akkermansia* transplantation can significantly increase the lifespan of Progeria mice and significantly delay the progression of neurodegenerative diseases. It has been shown that supplementation with the probiotic *Akkermansia* can increase the expression of tight-junction protein and ultimately improve the integrity of the intestinal barrier, while supplementation with DOFF happens to increase the abundance of *Akkermansia* in the gut of aging mice, which is one of the potential ways that DOFF improves cognitive function through the gut microbiome (Bu et al., 2021). Therefore, it is speculated that DOFF may improve the symptoms of intestinal microbiota disorder in elderly mice by regulating the intestinal microbiota.

The gut microbiota is the “second genome” of the human body, which plays a vital role in the development of immune organs, the health of the body, aging, and the occurrence of diseases. A large number of animal and human studies have shown that with the aging of the body, the composition of the intestinal microbiota will change, and the dynamic balance of the intestinal flora will be broken, which is manifested as a decrease in the diversity of microorganisms, a decrease in the number of *Bifidobacteria*, and an increase in the number of facultative anaerobes such as *Enterococci* (Du et al., 2021; Ling et al., 2022). Normal intestinal flora contributes to the integrity of the BBB, while dysbiosis may increase the permeability of the BBB. Some scholars have pointed out that flavonoids can significantly reduce the ratio of *Firmicutes/Bacteroidetes* (F/B) in the intestinal tract of mice, which was consistent with our experimental results (Zhang et al., 2022). In addition, some studies have found that reducing the F/B ratio in aging mice can alleviate the defect of microglial phagocytosis (Xu et al., 2023). At the genus level, it can be found that the relative abundance of *Akkermansia* versus *Muribaculaceae_unclassified* in the gut of the aging group is significantly reduced. Low concentrations of *Akkermansia* in the gut may indicate a thinner mucus layer and weakened intestinal barrier function, which can increase the translocation of LPS, causing inflammatory bowel disease, obesity, diabetes and even affecting neurological disorders LPS is able to induce the high production of inflammatory cytokines, which leads to the disruption of barrier permeability (Li et al., 2023). Experiments have shown that injecting LPS into mouse and human cerebral vascular endothelial cells can activate TLR4, and the activation of TLR4 in turn downregulates the tight junction protein so that circulating LPS can quickly flow from the blood into the brain, causing an inflammatory response (Pang et al., 2021). These results suggested that DOFF can increase the relative abundance of *Akkermansia* in the gut, increase the expression of tight junction protein ZO-1, reduce the production of inflammatory factors, inhibit the activation of microglia, effectively protect the structure of intestinal microbiota and intestinal barrier function, and alleviate the nerve damage caused by inflammatory response.

### Effect of DOFF on hippocampal neurons in aging mice

**FIGURE 5:**
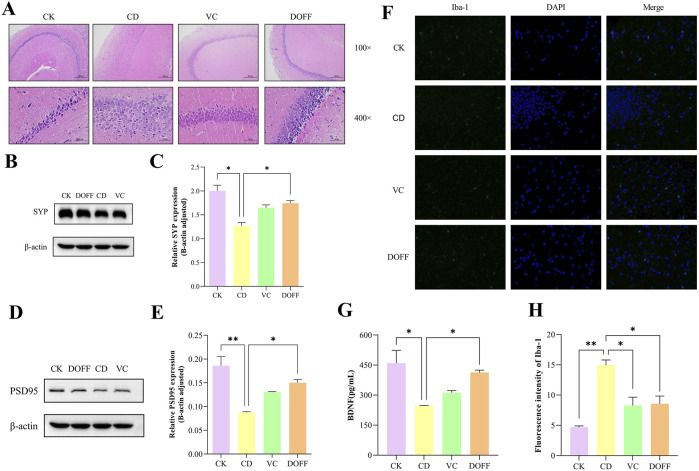
Effects of DOFF on neuronal damage, synapse-related protein expression, and brain microglial activation status in the brain of aged mice. **(A)** H&E staining of mouse hippocampus; **(B)** SYP levels measured by protein blotting; **(C)** quantitative detection of SYP protein bloting; **(D)** PSD95 levels measured by protein blotting; **(E)** quantitative detection of PSD95 protein bloting; **(F)** Expression of the microglial activati on marker Iba-1 in the hippocampus, representative images of immunofluorescence; **(G)** Hippocampal level of BDNF; **(H)** Fluorescence intensity of Iba-1. Data are presented as mean ± SEM, n = 15; *p < 0.05, **p < 0.01, ***p < 0.001, ****p < 0.0001, compared with the CD group.

The structure and function of neurons can reflect the degree of neurodegeneration. We performed pathological staining on mouse hippocampal tissues to observe the effects of DOFF intervention on hippocampal neurons through their histopathological changes. As can be seen from Figure 5A, compared with the CK group, the neuronal cells in the CD group were disordered and scattered, the nuclei shrank, and necrosis. It is worth noting that compared with the CD group, the morphology, and distribution of the hippocampus in the DOFF group were significantly improved, the number of neuronal cells increased, and the density of neurons in the hippocampus was significantly increased.

The main site of spatial learning and memory function in the brain is the hippocampus, where the destruction of neuronal and synaptic structures and the reduction of their number can cause memory and cognitive dysfunction. BDNF is a neurotrophic factor that promotes neuronal survival and differentiation and regulates synaptic growth and development, mainly in the hippocampus and cortex (Camuso et al., 2022). The expression of SYP and PSD95 proteins detected by Western blot is shown in Figures 5B–E. The protein expressions of SYP and PSD95 in the brain of mice in the CD group were significantly decreased and significantly increased after DOFF intervention (*p* < 0.05, *p* < 0.05). In addition, it is worth noting that the quantitative detection results of BDNF in Figure 5G showed that the BDNF content in the DOFF group was significantly higher than that in the CD group (*p* < 0.05). In conclusion, DOFF may regulate hippocampal synaptic plasticity by upregulating the expression of SYP and PSD95 in hippocampal tissues and increasing BDNF content, thereby improving cognitive impairment in mice.

Synaptic plasticity is produced by the continuous remodeling of synaptic connections in multiple regions of the brain, which is important for the development, learning, memory, and cognitive processes of the central nervous system, and is the basis for brain learning and memory (Magee and Grienberger, 2020). Changes in synaptic plasticity, such as decreased density of dendritic spines and decreased expression of synapse-associated proteins, can affect neuronal connections and impair neuronal function, leading to cognitive decline (Reza-Zaldivar et al., 2020). Studies have reported that aging mice often have changes in synaptic structure and reduced plasticity of synaptic function. SYP and PSD95 are two key proteins closely related to synaptic functional plasticity and changes in their expression levels are associated with enhancement or decline in learning and memory abilities (Wu et al., 2024). BDNF can bind to receptor-type tyrosine-protein kinase (TIB) to regulate the generation of immature nerve cells, the differentiation, development, and maintenance of neural precursor cells and nascent neurons, and can also participate in the formation, survival, maturation, and modification of synapses in mature neurons, as well as synaptic transmission and receptor activity. Both structural and functional aspects promote hippocampal plasticity and synaptic plasticity (Yi et al., 2024). In this study, D-gal-induced reduction of SYP and PSD95 protein expression levels and BDNF expression in the hippocampus of aging mice further demonstrated the reduction of synaptic plasticity. However, mice treated with DOFF showed increased expression of SYP and PSD95 proteins, as well as BDNF, which reflected significant improvements in synaptic structural and functional plasticity. These results suggested that DOFF has the potential to ameliorate aging-induced neuronal synaptic structural and functional impairment, especially in improving synaptic plasticity.

### Effect of DOFF on glial cell activation and Iba-1 protein expression in aging mice

A large number of microglia presented in the hippocampus, which plays a key role in the innate immune system. Studies have shown that long-term chronic activation of microglia can cause chronic neuroinflammation, leading to neuronal damage, which in turn leads to cognitive impairment (Zhang et al., 2024). To investigate the effect of DOFF on the degree of activation of microglia in the CA1 region of the hippocampus of senescent mice, we assessed it using immunofluorescence. Iba-1 was a green fluorescence marker (Figure 5F). Immunofluorescence results in Figure 5H showed that the green fluorescent cluster particles in the CD group were significantly higher than that in the CK group (*p* < 0.001), and the fluorescence intensity in DOFF group was significantly lower than that in CD group (*p* < 0.005). These results indicated that DOFF had a significant inhibitory effect on the activation of microglia.

In the process of aging, neuroinflammatory response is one of the main causes of cognitive dysfunction, especially in the hippocampus, which is more susceptible to inflammation (Ren et al., 2020). Aging microglia can influence cognitive processes by regulating neurogenesis, and neurogenesis is directly involved in the regulation of neuroplasticity. Neurogenesis was significantly reduced in aging mice, but with the elimination of microglia, neurogenesis as well as cognition and synaptic function were restored (Cornell et al., 2022). Microglia in a chronic aseptic inflammatory environment can further lead to the overproduction of pro-inflammatory cytokines and other cytotoxic mediators, which can have deleterious effects on neurons (Kempuraj et al., 2017).

### Effects of DOFF on inflammation and oxidative status in serum and hippocampal tissues of aging mice

**FIGURE 6:**
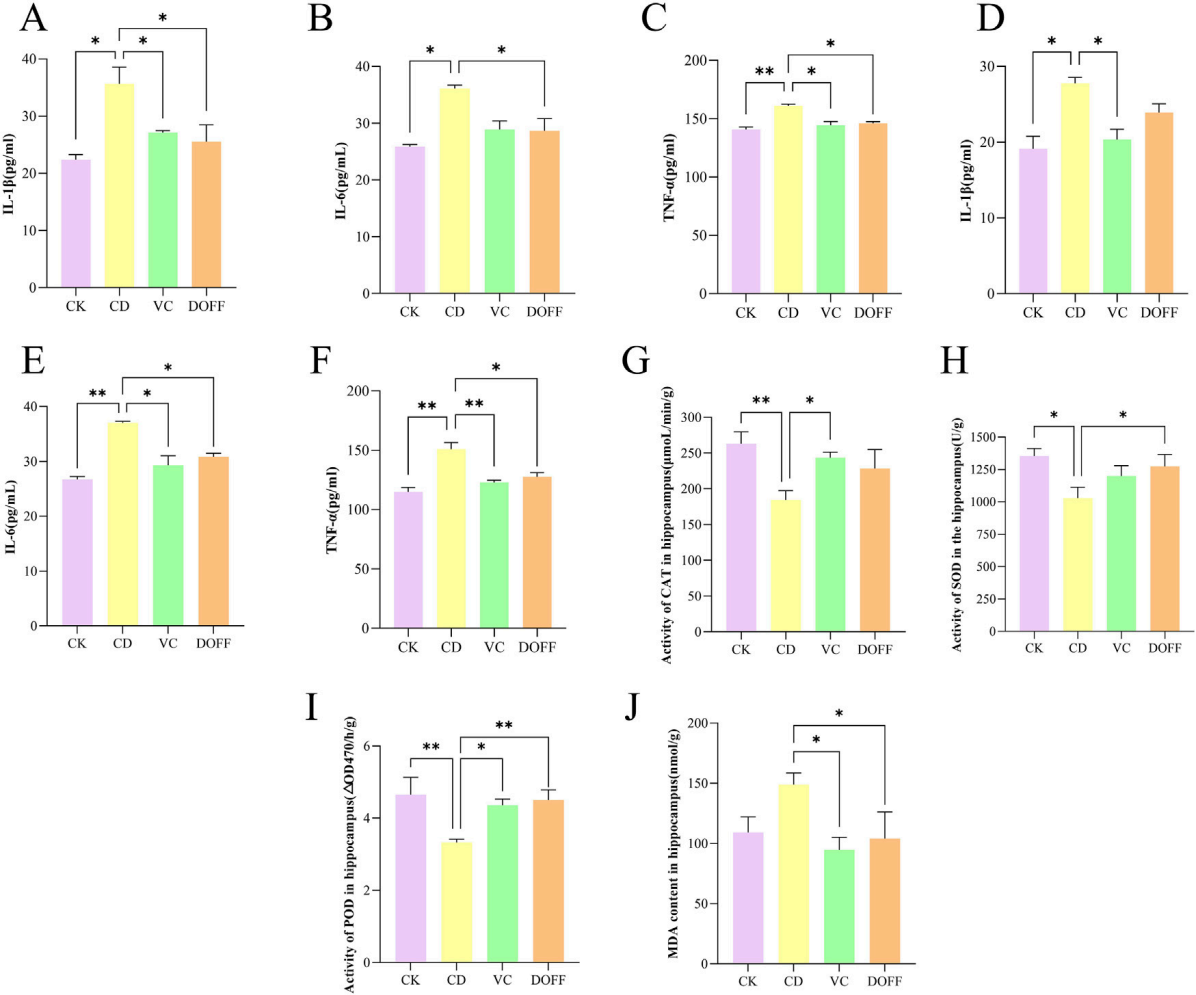
Effects of DOFF on inflammation and oxidative status in serum and hippocampal tissues of aging mice. **(A)** IL-1β activity in serum; **(B)** IL-6 activity in serum; **(C)** TNF-α activity in serum; **(D)** IL-1β activity in hippocampus; **(E)** IL-6 activity in hippocampus; **(F)** TNF-α activity in hippocampus; **(G)** CAT activity in hippocampus; **(H)** SOD activity in hippocampus; **(I)** POD activity in hippocampus; **(J)** MDA content in hippocampus. Data are presented as mean ± SEM, n = 15; *p < 0.05, **p < 0.01, ***p < 0.001, ****p < 0.0001, compared with the CD group.

Neuroinflammation plays an important role in the pathogenesis of cognitive dysfunction. The levels of TNF-α, IL-6, and IL-1β in serum and hippocampal tissues of mice were determined by ELISA. The results (Figures 6A–C) showed that the serum levels of IL-1β, IL-6, and TNF-α in the CD group were significantly increased compared with the CK group (*p* < 0.05, *p* < 0.05, *p* < 0.01). At the same time (Figures 6D–F), the levels of IL-1β, IL-6, and TNF-α in the hippocampus were also significantly increased (*p* < 0.05, *p* < 0.01, *p* < 0.01). Compared with the CD group (Figures 6A–C), the serum levels of IL-1β, IL-6, and TNF-α in the DOFF group were significantly reduced (*p* < 0.05, *p* < 0.05, *p* < 0.05). The levels of IL-6 and TNF-α were significantly reduced in hippocampus tissues (*p* < 0.05, *p* < 0.05) (Figures 6D–F). Prove it again, DOFF can inhibit the expression of pro-inflammatory factors and effectively alleviate the production of inflammatory factors *in vivo* and hippocampal tissues, thereby alleviating the inflammatory response.

Animal experiments have confirmed that the expression levels of reduced coenzyme II oxidase 2 (NOX2) and oxidative proteins in the brain of aging mice are higher after the onset of systemic inflammation, and the antioxidant defense capacity is lower, suggesting that modulating the inflammatory response and oxidative stress may be a potential way to stop cognitive decline during normal aging (d'Avila et al., 2018; Sharifi-Rad et al., 2022b). Studies have found that the increase in 1L-1β levels during aging can lead to impaired long-term potentiation (LTP), synaptic plasticity, and spatial memory of synapses in the CA1 region of the hippocampus, resulting in cognitive impairment (Sha et al., 2019). The results of immunofluorescence staining showed the abnormal activation of microglia induced by D-gal in senescent mice and the increased levels of inflammatory cytokines TNF-α, IL-6, and IL-1β in the hippocampus. Interestingly, after DOFF intervention, the number of microglia in aging mice decreased, inhibiting their activation, and the level of inflammatory factors in hippocampal tissue decreased, which ameliorated neuronal damage caused by neuroinflammation.

Oxidative stress is typical of aging (Liguori et al., 2018). The levels of MDA, SOD, CAT, and POD in the hippocampus of mice were analyzed. As shown in Figures 6G–I, the activities of SOD, CAT, and POD in the CD group were significantly reduced compared with the CK group (*p* < 0.05, *p* < 0.01, *p* < 0.01). Compared with the CD group, the activities of SOD and POD in the DOFF group were increased (*p* < 0.05, *p* < 0.01). The level of MDA in the DOFF group was significantly lower than that in the CD group (*p* < 0.05, Figure 6J), and the decrease in MDA level also reflected the improvement of lipid peroxidation in the cell membrane. These results suggest that DOFF can effectively improve oxidative stress damage caused by aging by inhibiting the production of MDA and increasing the activity of SOD, CAT, and POD in aging models.

SOD, CAT, POD, and MDA are the main markers reflecting the redox ability of the body to scavenge oxygen-free radicals (Liguori et al., 2018). To verify whether the anti-aging effect of DOFF is related to antioxidant activity, SOD, CAT, and POD activities and MDA content in mouse serum were detected. The results of this study showed that the activities of SOD, CAT, and POD in aging mice decreased and the content of MDA increased, suggesting that the ability of mice in the aging group to scavenge oxygen free radicals decreased. Combined with the above results, the number of hippocampal mature neurons and the expression of synapse-related proteins decreased in the aging group, which suggested that D-gal may lead to neuronal death and synaptic plasticity by weakening the oxygen radical scavenging ability, resulting in cognitive impairment in mice. After DOFF intervention, the relevant indexes were positively adjusted, the activities of SOD, CAT, and POD were improved, the content of MDA and the oxidative stress response *in vivo* was reduced, the hippocampal mature neurons were protected, and the expression of synapse-related proteins was increased, thereby improving cognitive impairment.

### Effects of DOFF on cognitive ability in aging mice

**FIGURE 7:**
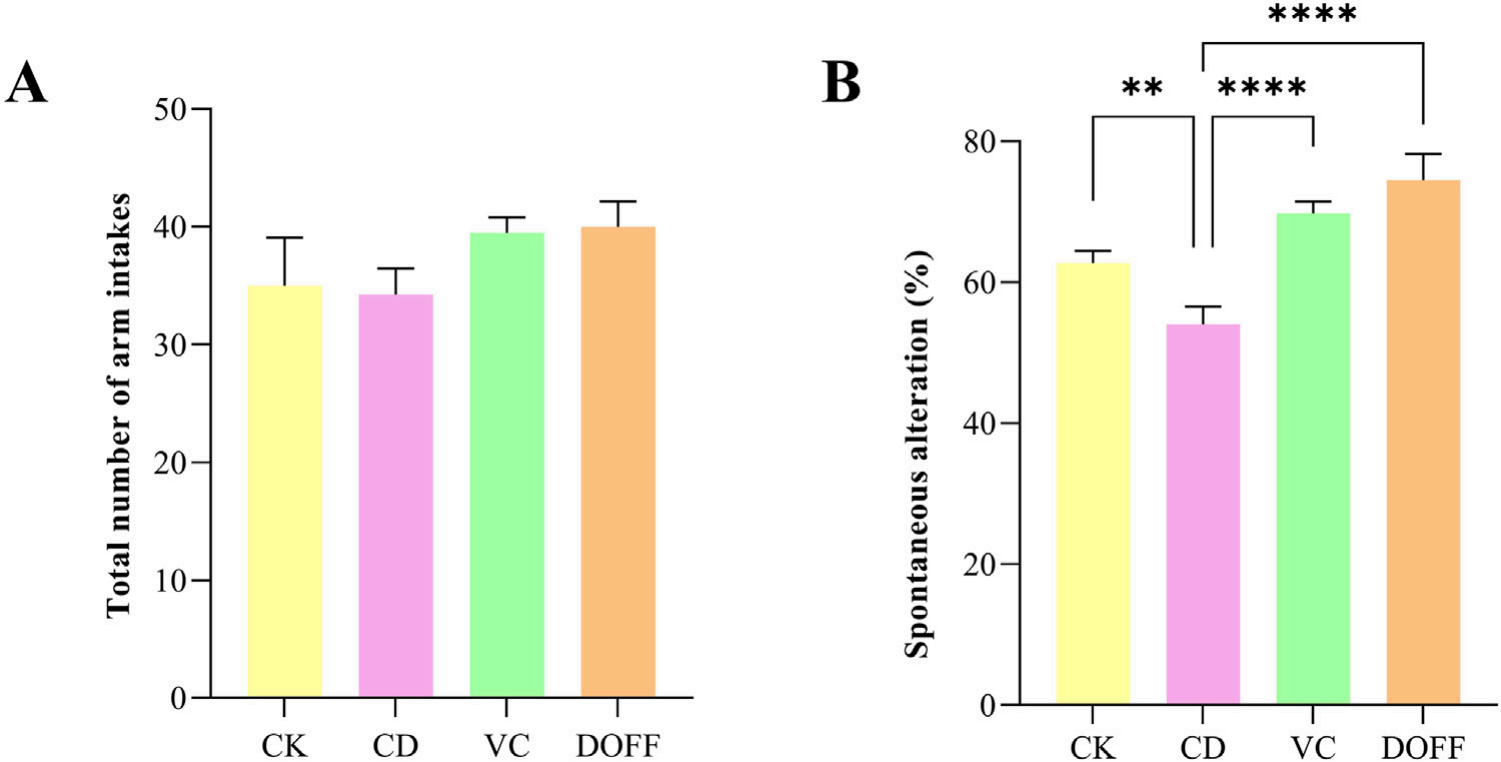
Y maze test. **(A)** The total number of arm entries, **(B)** Spontaneous alteration Data are presented as mean ± SEM, n = 15; *p < 0.05, **p < 0.01, ***p < 0.001, ****p < 0.0001, compared with the CD group.

In the Y maze test, the total number of arm entry reflected the mice’s autonomous activity ability, and the result of spontaneous alternation correct rate could reflect the mice’s autonomous exploration and short-term working memory ability. As shown in Figure 7A, there was no significant difference in the total number of arm insertion of the three groups of mice (*p* > 0.05), and there was no significant difference in autonomous activity ability; Compared with CK group, the correct rate of spontaneous alternation in CD group was significantly reduced (*p* < 0.0001) (Figure 7B), indicating that spatial memory ability of aging mice declined, while intervention in VC group and DOFF group could significantly alleviate this phenomenon (*p* < 0.0001). Through the animal behavior experiments related to learning and memory cognition, it was found that DOFF enhanced the exploration desire and short-term working learning and memory ability of aging mice, and improved the cognitive function of aging mice to a certain extent. In our previous studies, it revealed that *D. officinale* could reverse the levels of metabolites derived related to cognitive function improvement, and these metabolites were closely associated with the key microbiota (Sun et al., 2022; Xu et al., 2023).

## Conclusion

Taken together, our findings suggested that D-gal-induced mouse models of aging can lead to neuronal damage as well as neuroinflammation, accompanied by gut microbiota disorders, intestinal barrier damage, and LPS exudation. DOFF intervention can positively regulate the composition of intestinal microbiota in D-gal mice, strengthen the integrity of the intestinal mucosal barrier, and alleviate intestinal barrier injury. It also improved cognitive dysfunction by reducing oxidative stress and inflammation, increasing synaptic plasticity, and up-regulating BDNF levels. At the same time, DOFF supplementation can inhibit the activation of microglia induced by the excessive production of inflammatory factors, and improve neuroinflammation and neuronal morphological damage.

## Data Availability

The original contributions presented in the study are included in the article, further inquiries can be directed to the corresponding authors.
